# Two Sorts of Waste of Children

**Published:** 1917-04-07

**Authors:** 


### SOME PROBLEMS OF TO-DAY.*

### II. Two Sorts of Waste of Children.

It was shown in a former article that we in this country made a national admission of every child's right to be fitted by education for life nearly half a century before we admitted his prior right to life itself. One of the most hopeful outcomes of the war is surely that demand for the full realisation of these two great rights which comes not now from any superior class trying to "raise the people," but from within the democracy itself. For instance, among the many educational resolutions submitted in January to the Labour Conference was one from the Boilermakers' Union, which demanded that municipal bodies should be obliged by law to take speedy and efficient measures by the provision of mother-schools, health visitors, supervision of milk and other food supplies, and school clinics, to secure that "every child, no matter how lowly born, may receive such care and attention as to give it the best possible chance to survive the infant ailments which every day rob the nation of so many precious lives." A more powerful body, the Workers' Educational Association, which federates 2,150 working-class bodies, such as trades unions and co-operative associations, has put forth a most carefully considered system covering every need of a child from the first day to the last of organised schooling. One of its first demands is for nursery schools, small and plentiful, so as to be easily reached, for infants from two to six, development and care being more the object at this stage than instruction. We hear—again—of a roused demand among agricultural labourers, the most "old-fashioned" of all classes. A Dorsetshire private guarding German prisoners lately owned to a regretful envy of their acquirements.

All the suggested programmes, therefore, have two leading ideas: first, to remedy the wastage by death and damaged health of our infant citizens; and, secondly, to remedy the pitiable loss and waste made by half-educating the young and then turning them loose upon our streets till such time as they are old enough for employment. In older days we did not do this. The gentleman's son and daughter, as childhood ended, became page and bower-maiden in great house or court, and had regular school lessons as well as organised duties and training. The poor man's son was apprenticed, even his daughter often went from home for training; the arts and crafts were properly taught. The notes to our Church Catechism show that masters had to see that 'prentices learnt their religion. One old schoolbook describes itself as specially handy for reading lessons in the workshop. But our "Board-school" education ended just about the time when, if we have been well grounded, we become capable of really learning! Accordingly, now we have the demand, voiced all over the country, that in one way or another those plastic years shall be used. Before the war the crush of unskilled labour caused many lamentable sights. The idle "corner-boy" who knew no trade and was degenerating into loafer and perhaps thief had very probably found himself work as an errand-boy or van-boy till ousted by a younger boy when too old. The crowding of girls into bottle-washing and other unskilled employments kept wages down to below any living wage, and filled the poorer districts with women imploring charwork. It is certain that something will be done to alter this condition, and to cure the evils it breeds. We think, for instance, of a burglar as a powerful and desperate ruffian. Some seven-eighths of those convicted in late years have been boys under twenty-one.

Many questions of detail will have to be settled, of course. How far, for instance, can young people who have to begin to earn as soon as possible be spared in their employers' time to gain knowledge? If the employers do so spare them, will they get an adequate return in better work done? One firm who already give a practical answer to this question are Messrs. Crosse and Blackwell, who allow their younger girl employees to stop work at four, go to a good tea in their own club-room, and then receive not less than 7½ hours a week of lessons from teachers sent from a Women's Institute of the London County Council.

A further question arises. Could not these young people, whose efficiency and rate of wages depend so much upon their acquired skill, receive at this time what educationists call *vocational teaching*? This question is a very pressing one, and is being vigorously debated. For instance, the Child-Study Society are making it the subject of a whole course of discussions, the first of which was opened on March 8 by Mr. Coffin, Director of Education at Bradford, who went wholeheartedly for the idea of some specialisation. He urged, as others have done, that many young people who have not been "good at their books" gain entirely new application when their work is seen as a means to an end. Whereas a statute of 1563 made apprenticeship compulsory, now only about 7 per cent. of journeymen have served in this way. Instead of boys and girls being "learners through doing," they have become independent workers at easy tasks, which are blind-alleys. The masses must be hand-workers, and greater skill will be needed in the competition of nations. To prepare *for life* is the admitted purpose of education; will not this be best gained also by "vocational teaching"? Some corporate life, some physical development, some general study to broaden the outlook should save from narrowness.

Now the opponents of this theory always argue that in childhood "a good general education" without specialising should be laid as a foundation for technical work. Their view was set forth with vigour and with emotion at another discussion (March 29) of the Child-Study Society by the Principal of the Shoreditch Technical Institute, who pleaded for the admission of those whose bread is earned by hand-work, "the lad who makes the chair," into the precious heritage of English Literature. What, he asked, does a boy need to know in order to realise his place as a responsible citizen and human being? Surely "The Humanities," what man has done, what man has made, what man is becoming. But we have been prone to teach Alfred and his cakes and the marriages of Henry VIII. as national history, and no word of Wren or Inigo Jones. His own boys had been questioned on violets and cheese as products of Parma when he had been trying to teach them the making of Italy. Even industrial history is the story of man's hopes, labours, ambitions, despairs; it must not be reduced to dates of the passing of Acts of Parliament. The perils which he

* The previous article appeared on March 31, p. 523.

saw to wait on vocational training were this cramping of the mind, the difficulty of changing the vocation if found unsuitable, and that of preventing the child from being exploited as an earner of dividends. At present, however, only one boy in forty in London enters a trade school, the girls being rather more numerous. The present writer would urge that in their case it should not be forgotten that, though she may incidentally be a teacher or an artist, a girl's vocation is to be a woman. Child-bearing cannot be deputed, and even if the Utopian theory of child-rearing by experts should be adopted, the experts will be women. Neither general nor vocational education which fails to reckon with this truth can succeed.

